# Supplementary material for: Effect of Input C/N Ratio on Bacterial Community of Water Biofloc and Shrimp Gut in a Commercial Zero-Exchange System with Intensive Production of Penaeus vannamei
Source: Microorganisms. 2022 May 20;10(5):1060. doi: 10.3390/microorganisms10051060 (PMC9146922; doi:10.3390/microorganisms10051060)
Supplement: Supplementary file 1 [file microorganisms-10-01060-s001.zip › microorganisms-1716296-supplementary.pdf]

Supplementary Materials

Supplementary Table S1

The main water quality characteristics of the shrimp nursery pond.

Parameter	Value
Salinity (g L^{-1})	28.3
Water temperature (°C)	29.4
Dissolved oxygen (mg L^{-1})	5.6
pH	7.5
Alkalinity (mg L^{-1} as $CaCO_3$)	194
Total ammonia nitrogen (mg L^{-1})	2.8
Nitrite nitrogen (mg L^{-1})	1.5
Nitrate nitrogen (mg L^{-1})	31.3
Total nitrogen (mg L^{-1})	57.1
Biofloc volume (mL L^{-1})	11.5
Volatile suspended solids (mg L^{-1})	284.2

Supplementary Table S2

The overall means (minimum, maximum) of selected water quality parameters from biofloc-based tank systems during an 8-week intensive production trial of *P. vannamei* with three input C/N ratios.

Parameters	CN8	CN12	CN16
Salinity (g L^{-1})	27.6 (25.0, 29.1)	27.4 (24.7, 29.1)	27.1 (24.2, 29.1)
Temperature (°C)	28.6 (25.2, 31.3)	28.5 (25.2, 31.5)	28.8 (25.4, 31.9)
DO (mg L^{-1})	6.1 (5.3, 7.3)	6.2(5.2, 7.4)	5.9 (5.0, 7.1)
pH	7.5 (7.0, 7.8)	7.5 (7.0, 7.8)	7.4 (6.9, 7.8)
Alkalinity (mg L^{-1} as $CaCO_3$)	168.4 (132.0, 210.0)	183.6 (132.0, 225.0)	214.3 (132.0, 241.0)
TAN (mg L^{-1})	0.42 (0.04, 1.23)	0.33 (0.02, 1.01)	0.27 (0.01, 0.93)
NO_2^--N (mg L^{-1})	0.32 (0.08, 1.26)	0.46 (0.10, 1.71)	0.53 (0.02, 2.23)
BFV (mL L^{-1})	17.7 (9.5, 27.8)	20.9 (9.5, 26.3)	22.7 (9.5, 29.5)
VSS (mg L^{-1})	201.9 (123.0, 292.5)	217.5 (135.0, 303.7)	233.6 (134.0, 321.0)

DO: Dissolved oxygen; TAN: total ammonia nitrogen; BFV: biofloc volume; VSS: volatile suspended solids.
